# The State Medical Examining and Licensing Board1Extract from the President’s Inaugural Address, delivered in Albany, Feb. 3, 1891, at the opening of the Eighty-fifth Annual Meeting of the Medical Society of the State of New York.

**Published:** 1891-04

**Authors:** William Warren Potter

**Affiliations:** Buffalo


### THE STATE MEDICAL EXAMINING AND LICENSING BOARD.[1]

By WILLIAM WARREN POTTER, M. D., of Buffalo.

. . . . . . . . . .

It is the written law of this Society that the president shall, at the opening of each annual session, make to it a communication setting forth the condition of the medical profession in the State, and make such suggestions in relation to its improvement as may be deemed appropriate.

It is a fortunate circumstance that, at this time, there is in the condition of the profession so much to commend and so little to criticise. If we have not attained that degree of perfection which we could desire, if we have not yet reached the point where we can quit the ocean of endeavor and lie down by the still waters of Utopia, it is in part because we are human, and in other part because this is a progressive world, and its purposes are accomplishing themselves in broader and ever widening circles.

Nevertheless, it is a satisfaction that we are able to note some progress year by year towards that standard of medical excellence which, when attained, will enable our profession to take the rank which our supremacy as a Nation demands, and which our pride as a Commonwealth warrants. The contribution of this Society for the year 1890, looking to that end was

### A STATE EXAMINING AND LICENSING BOARD, which may be regarded very properly as the most important step taken by this State within the range of recent years in the direction of medical educational reform, and it signals with a good omen the beginning of the last decade of the Nineteenth Century.

For a period of time reaching back to the early days of this Society, the question of improvement in our methods of medical teaching and licensing has, in one way and another, been prominent at its annual meetings, and for twenty or thirty years past, the presidents of this body, with but few exceptions, in their annual addresses have laid emphasis upon the importance of some radical change in the prevailing system. There has been scarcely a year from 1824 down to the present, that the question has not come up in some form or another, either in debate or in formal address, so

1. Extract from the President's Inaugural Address, delivered in Albany, Feb. 3, 1891, at the opening of the Eighty-fifth Annual Meeting of the Medical Society of the State of New York.

that year by year the note of warning has been sounded on the subject of separating the teaching and licensing powers. More definite shape, however, was given to the matter some seven or more years ago, when the Committee on Legislation of the Medical Society of the County of Erie reported a plan for the complete separation of the "unfortunate, unwise, and debasing co-partnership of the educating and licensing powers." This was in the nature of a carefully drawn bill, which, after receiving the approval of the Erie County Society, was submitted to the several county medical societies throughout the State, and received the commendation of four-fifths of those societies. It was then presented to this Society through its legislative committee where it was also approved, and by this latter committee was presented to the Legislature.

It would be a waste of time to recount the various steps taken, and the ups and downs of the measure in its parliamentary struggles through both this Society and the several legislatures from 1884 onward, until finally the present law was evolved by the Legislature of 1890, receiving the approval of the Governor on June 5th of the same year. Suffice it to say, that its history during those seven long years of waiting is the oft-repeated tale of trials and triumphs, of defeats and disappointments; but finally the hour of rejoicing came, and we are now apparently near the realization of our long desired deliverance from the thraldom of a pernicious system which has retarded the progress of medical science in this State more than all other causes combined.

If the present law is an apparent modification of the original bill which this Society offered to the legislature, who will dare assert that it is not a better one? It is true the original thought prevalent in the minds of most of us was to obtain a single mixed board; whereas the law contains provisions for three separate boards, representing the three several state medical societies recognized by the statutes of the State of New York. But let it be remembered that although there are to be three boards, they are limited to a *single standard* in all the important and fundamental departments of the curriculum of medicine; and, moreover, there is to be but a single licensing body, namely, the Board of Regents of the University of the State of New York. Finally, the Medical Colleges are all bound, by the provisions of this law, to give three full courses of medical instruction in different years before granting diplomas, since physicians will not be admitted to the examination for license with less preparation. And who can justly asseverate that this is not a better solution of the problem than the plan first proposed?

It may be that our friends, the enemy — an enemy that appealed to all the weaker qualities of human nature to thwart our purpose, and who struck hands with any- and everybody who would aid in our defeat—it may be, I repeat, that they builded better than they knew, and that this Society has been placed in a stronger position than it would have been possible without such opposition. At all events, let us accept the law in good faith, sustain its provisions considerately and loyally, do all in our power to strengthen the hands of the Board of Regents which is charged with its execution, and then we shall enjoy the proud consciousness of having performed well our part in the great drama of medical progress.

If, on due trial, perchance there shall be found defects in the law, these can be remedied easily when experience shall point them out; but, as a final word on this subject, let me urge upon this Society the importance of standing as a unit against any and all attempts to modify its present provisions or to break its full effect and force until it is thoroughly tested. Verily, let us love this law for the enemies it has made.

The first section of the law, "To Establish Boards of Medical Examiners of the State of New York for the Examination and Licensing of Practitioners of Medicine and Surgery; and to further regulate the Practice of Medicine and Surgery" in this State provides that there shall be an examining board of seven members, representing the Medical Society of the State of New York; that the power to appoint this board shall vest in the Regents of the University of the State of New York; that such appointments shall be made from a list of nominees to be submitted from this Society; and that the number of such nominees shall equal or exceed twice the number of appointments to be made. The first section of the law further provides that "each of said nominees shall be nominated by a majority vote at the annual meeting of the society with which said nominee may be in affiliation, and the names of the persons so nominated shall be transmitted before the first day of July, 1891, to the said Board of Regents, under the seal of and signed by the president and secretary of the society so nominating."

From this it would appear that this Society must, at this session, nominate a list of fourteen or more names to the Board of Regents, and that such nominations must be made by vote. While it must be conceded that it is of the first importance that the men who are to compose this board must possess the highest character, ability, and fitness for the service required, and that the interests of all parts of the State should be conserved in their selection, yet, for obvious reasons, it is equally important that this duty shall be performed in a manner that shall interfere as little as possible with the scientific work of the Society. It is to be hoped, therefore, that some method may be evolved that will wisely, considerately, and adequately fulfill all the various requirements of this important function that the law has placed upon our shoulders.

To this end your patient efforts are earnestly invoked.

